# Detecting Distal Radius Fractures Using a Segmentation-Based Deep Learning Model

**DOI:** 10.1007/s10278-022-00741-5

**Published:** 2022-12-21

**Authors:** Turkka T. Anttila, Teemu V. Karjalainen, Teemu O. Mäkelä, Eero M. Waris, Nina C. Lindfors, Miika M. Leminen, Jorma O. Ryhänen

**Affiliations:** 1 Musculoskeletal and Plastic Surgery, Department of Hand Surgery, University of Helsinki and Helsinki University Hospital, Topeliuksenkatu 5B, Helsinki, 00260 Finland; 2 Department of Orthopedics, Traumatology and Hand Surgery, Central Finland Hospital, Jyvaskyla, Finland; 3 Medical Imaging Center, Radiology, University of Helsinki and Helsinki University Hospital, Helsinki, Finland; 4 Department of Physics, University of Helsinki, Helsinki, Finland; 5 Analytics and AI Development Services, IT Department, Helsinki University Hospital, Helsinki, Finland; 6 Department of Otorhinolaryngology and Phoniatrics, University of Helsinki and Helsinki University Hospital, Helsinki, Finland

**Keywords:** Fractures, Artificial intelligence, Deep learning, Radius fractures, Diagnostic tests

## Abstract

Deep learning algorithms can be used to classify medical images. In distal radius fracture treatment, fracture detection and radiographic assessment of fracture displacement are critical steps. The aim of this study was to use pixel-level annotations of fractures to develop a deep learning model for precise distal radius fracture detection. We randomly divided 3785 consecutive emergency wrist radiograph examinations from six hospitals to a training set (3399 examinations) and test set (386 examinations). The training set was used to develop the deep learning model and the test set to assess its validity. The consensus of three hand surgeons was used as the gold standard for the test set. The area under the ROC curve was 0.97 (CI 0.95–0.98) and 0.95 (CI 0.92–0.98) for examinations without a cast. Fractures were identified with higher accuracy in the postero-anterior radiographs than in the lateral radiographs. Our deep learning model performed well in our multi-hospital and multi-radiograph system manufacturer settings. Thus, segmentation-based deep learning models may provide additional benefit. Further research is needed with algorithm comparison and external validation.

## Introduction

Distal radius fractures (DRFs) account for up to 20% of all fractures in a typical emergency department [1, 2]. Diagnosis and treatment are based on clinical examination and correct interpretation of radiographs. Misinterpretation of radiographs is common [3–5] and is also a reason for litigation [6]. A reliable deep learning (DL) model would be an invaluable aid in urgent emergency department conditions to reduce misdiagnosis.

During the last decade, machine learning and its subclass deep convolutional neural networks (CNN) have excelled in image recognition and segmentation tasks [7]. Images can be analyzed using object detection and semantic segmentation techniques, which differ in features [8]. The selection of an optimal approach and CNN can be difficult and is dependent on the task and usable data. A wide range of orthopedic trauma radiographs have already been investigated in several studies but mainly using object detection [9–12].

For this study, we developed a segmentation-based U-net [13], which uses the pixel-level annotations of fractures rather than a box annotation, DL model to detect DRF from radiographs. The proposed segmentation approach allows the network to precisely indicate which features (at the pixel level) it uses for fracture detection without resorting to class activation mapping or other indirect model interpretation techniques. This has a benefit of providing higher levels of confidence in the predictions, which is essential for medical applications.

The aim of this study was to validate this DL model and to test the feasibility of this approach.

## Materials and Methods

### Data Acquisition

A cohort of consecutive adult (≥ 18 years) wrist trauma patient radiographs from six hospital emergency rooms from 2016 was acquired from the Helsinki University Hospital’s Picture Archiving and Communication System (PACS). Radiographs were subsequently stored in the hospital electronic database (HUS Datalake).

The radiographs were acquired using radiograph systems from nine different manufacturers, namely, Samsung Electronics (49%), Fujifilm Corporation (22%), Philips Medical Systems (16%), Canon Inc. (10%), Agfa (2%), GE Healthcare (1%), Carestream Health (0.2%), Siemens (0.1%), and Kodak (0.05%). The percentages indicate the proportion of images taken with the respective manufacturer’s systems. The DICOM files were converted to NIFTI files with lossless conversion and pseudonymized.

In case of multiple projections, a hand surgery (subspeciality) resident (T.A.) identified and included two projections of radiographs that were closest to the true postero-anterior and lateral projections. Radiographs focused on hand or forearm and wrists with previous wrist arthrodesis, severe wrist osteoarthritis, or open physis were also excluded. Radiographs with casts were included.

**Fig. 1:**
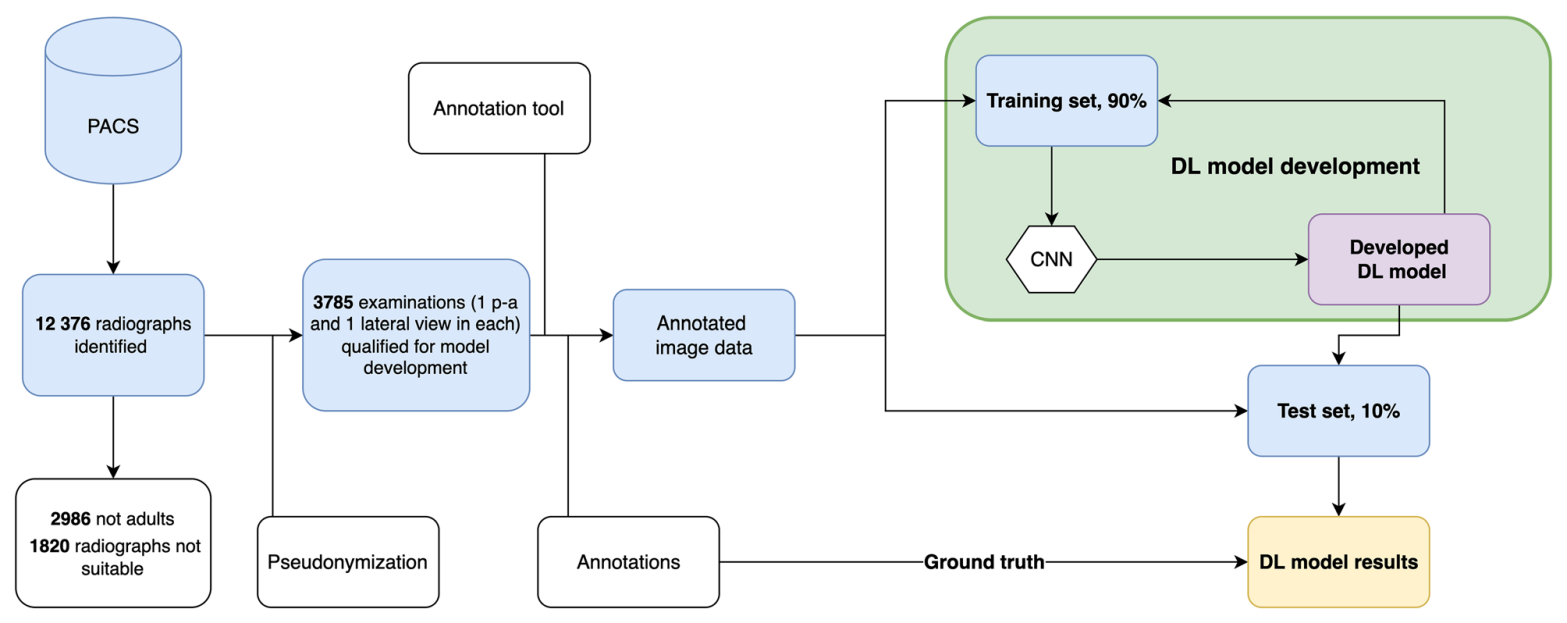
Data retrieval, annotation, and analysis process. PACS = picture archiving and communication systems; p-a = postero-anterior; CNN = convolutional neural network; DL model = deep learning model

To develop and test the performance of an algorithm, the radiographs were randomly split patient-wise into training and test sets (10% for the test set). Data selection details and the overall workflow of the conducted study are shown in Fig. 1.

### Annotation

**Fig. 2:**
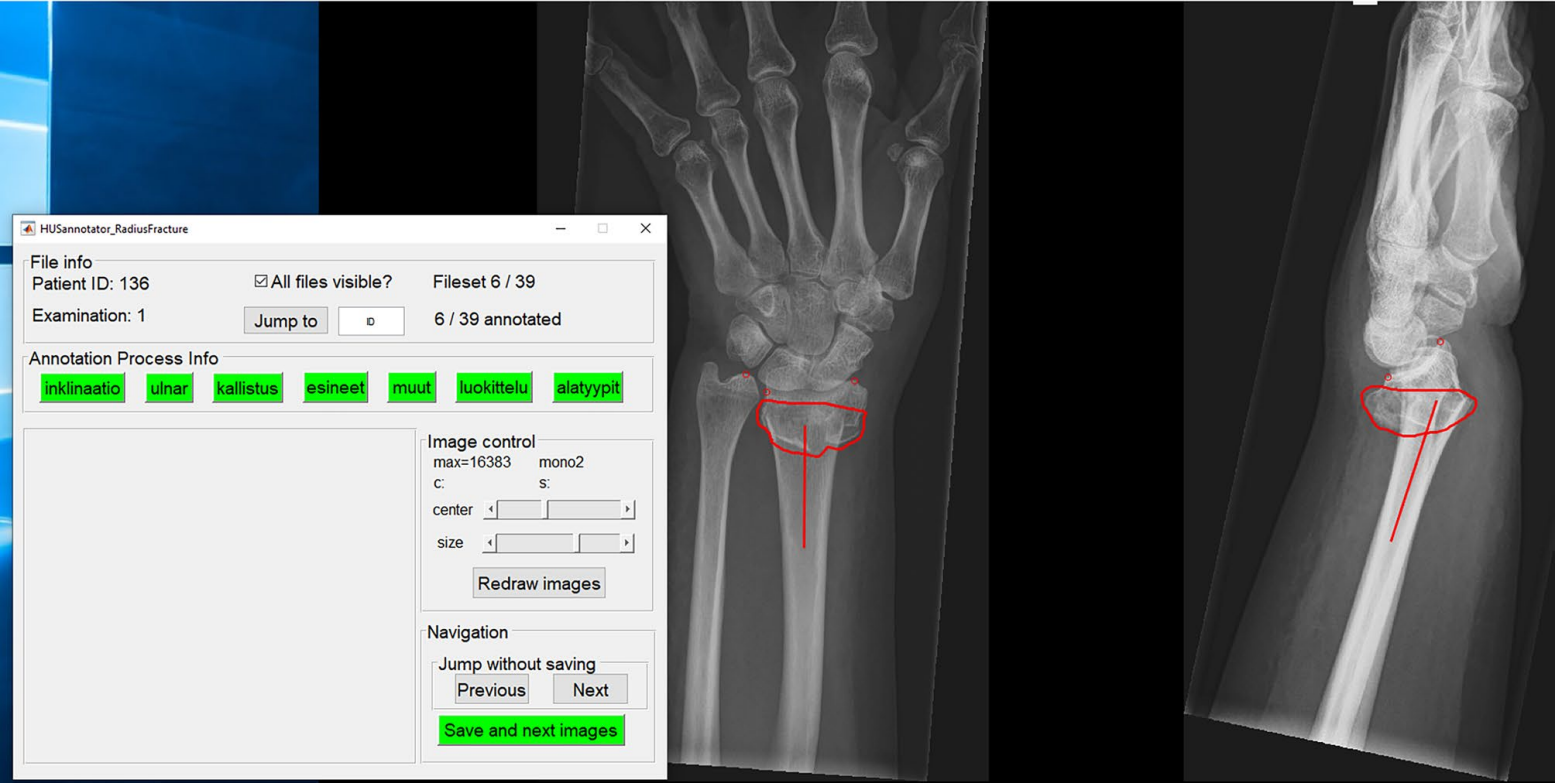
An example of the annotation software. The red outline surrounding the fracture area was used for DL model development. “Annotation process info” box texts in English: inklinaatio = inclination; ulnar = ulnar variance; kallistus = articular tilt; esineet = foreign objects; muut = other remarks; luokittelu = classification; alatyypit = subtypes/drawings

An in-house engineered image annotation software was developed to enable clinicians’ efficient annotation of the radiographs (see Fig. 2). The tool was developed as standalone MATLAB application with a graphical user interface (The MathWorks. Matlab version 9.4. Natick, Massachusetts: The MathWorks, Inc., USA; 2018).

The annotation process was performed in a setting where background information about the patient or diagnosis history was not available to the annotator. The developed tool allowed the annotator to adapt the following contrast settings: dynamic range (scale from black to white), window size, and center point to optimize a visual inspection.

A total of 3785 examinations, each comprising one postero-anterior and lateral projection, were included in the study. In the annotation process, the fracture location (when present) was annotated on the radiographs in both projections. The presence of a cast was also annotated (yes/no). One hand surgery resident (T.A.) with 5 years of training annotated the radiographs for the training set (90%). Three consultant hand surgeons (E.W., N.L., and J.R.) with an average experience of 18 years independently annotated the test set (10%). In case of disagreement, consensus was reached through a live adjudication session.

To assess the reliability of annotation, the kappa coefficient for interrater reliability was calculated between the resident’s and three hand surgeons’ consensus test set assessment. The resident and the three consultants assessed a set of 30 examinations again 6 weeks after primary annotation.

The inter-observer reliability between the resident (T.A.) and consensus of three consultants was 0.98. There was a disagreement between the consultant’s independent assessment in 27/386 (7%) of examinations of 18/249 patients. The intra-observer reliability was 1.0 for the resident and 1.0 for the consultants.

The positive and negative likelihood ratios were calculated for the internal test set radiographs without a cast. To calculate the post-test probabilities, a pre-test probability of 0.47 was used based on the incidence in the set.

The training and test set demographics are shown in Table 1. In the test set, four radiographs taken with cast did not have a fracture.

**Table 1:** Training and test set demographics

	Patients	Examinations	Gender female (%)	Cast (%)	Fracture (%)	Age, years, median (range)
Training set	2388	3399	61*	44%	69	60 (18–100)
Test set	249	386	67^a^	46%	70	61 (18–90)

*Gender data not available for 15 patients

^a^Gender data not available for 1 patient

### Data Preprocessing

The radiographs were first resized to 0.1 × 0.1 mm^2^ resolution and underwent contrast-limited adaptive histogram equalization [14], an approach similar to the study by Pan et al. [15]. Finally, the image intensities were normalized to a 0–1 range prior to feeding to the CNN.

### CNN Architecture

We chose a segmentation-based approach for the DRF detection. We trained a variant of the extensively adopted U-net architecture with 25 layers using manually drawn fracture locations as training targets [16]. A single postero-anterior or lateral view was fed into the network, and a confidence value (probability) for the presence of a fracture was produced for each pixel (see detailed description in the Supplement).

### Testing

The model produced fracture confidence values for each image pixel. We recorded the maximum confidences in each radiograph and compared these against the ground truth of fracture present in the radiograph or not. We chose the final decision threshold used in the performance metric calculations by maximizing the fracture detection accuracy in the validation data; the optimal cutoff for the network output confidence was ≥ 0.61. The CNN was implemented in Keras and Tensorflow version 2.0 [17, 18].

### Model Evaluation

For the test set, we used the three consultants’ consensus as the ground truth and calculated the sensitivity, specificity, accuracy, negative predictive value (NPV), and positive predictive value (PPV) with 95% confidence intervals (CI). To assess test discrimination, we used receiver operating characteristic (ROC) curve analysis and calculated the area under the curve with 95% CI bootstrapping 10^5^ samples. We calculated the ROC curves by varying the segmentation network’s confidence threshold. The postero-anterior and lateral views were evaluated both separate and in unison.

## Results

We detected 262 out of 271 examinations with a DRF from a total of 386 examinations in the test set. The area under the ROC curve was 0.97 (CI 0.95–0.98) and 0.95 (CI 0.92–0.98) for examinations without a cast.

**Fig. 3:**
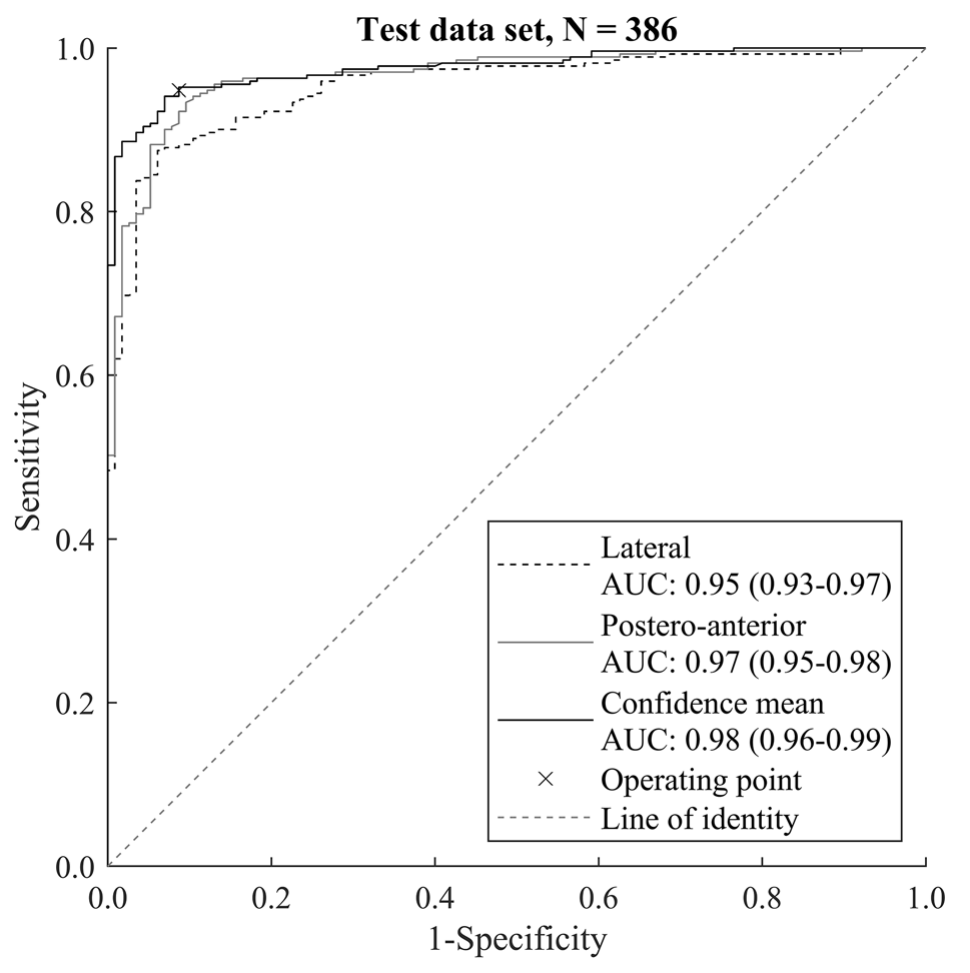
Receiver operating characteristic curves showing the algorithm’s discrimination performance on the test set. The pre-determined operating point (×) is close to the upper-left corner, which shows that the model performs well in the test set data and is well balanced

**Fig. 4:**
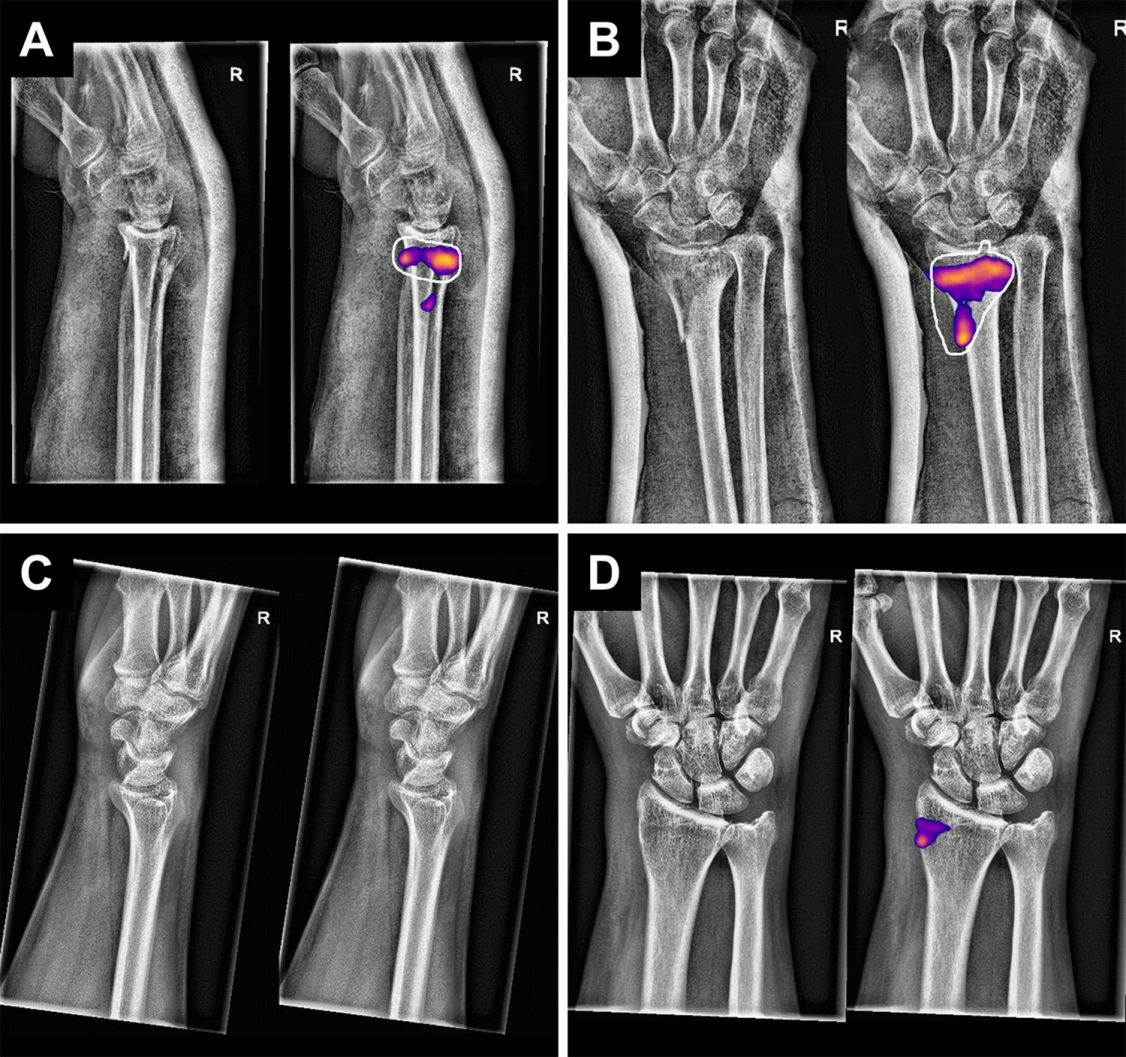
The top two images (**A**, **B**) show an example of the algorithm’s true positive fracture predictions, where the white outline shows the free drawn manual labeling considered the ground truth. In the bottom two images (**C**, **D**), no fracture is detected in the lateral view (C) but the proximal radial styloid process is incorrectly indicated as fractured in the postero-anterior view (**D**). The color overlay is produced automatically by the segmentation tail of the network, precisely describing which part of the image the model predicts having a fracture

For DRF detection from individual radiographs, the area under the ROC curve was 0.96 (CI 0.94–0.97) and 0.94 (CI 0.91–0.96) for radiographs without a cast. Fractures were identified with higher accuracy in the postero-anterior radiographs than in the lateral radiographs. For radiographs without a cast (a typical clinical scenario where the model is applied), the accuracy for fracture detection in the lateral and postero-anterior radiographs was 0.85 and 0.90, respectively. The ROC curve is presented in Fig. 3. Figure 4 shows examples of a correct DRF detection and an incorrect assessment by the DL model.

See Table 2 for confusion matrices for individual radiographs and for examinations separately. In Table 3 are presented the results of the test set for radiographs with and without a cast separately.

**Table 2:** Confusion matrices for individual radiographs where postero-anterior and lateral views were considered separately (on the left) and for examinations (1 postero-anterior and 1 lateral view) where the decision was based on the mean neural network confidence of the two views (right). Abbreviations: p-a = postero-anterior; lat = lateral

**Single radiographs**		Predicted			**Examination (1 p-a and lat)**		Predicted		
Actual		Fracture	Normal		Actual		Fracture	Normal	
	Fracture	499	43	542		Fracture	262	9	271
	Normal	27	203	230		Normal	23	92	115
		526	246	772			285	101	386

**Table 3:** Results for the test set with 95% confidence intervals

	All radiographs	Lateral projection only	Postero-anterior projection only	Either lateral or postero-anterior above threshold	Lateral and postero-anterior confidence average
	772 radiographs	386 lateral	386 p-a	386 examinations	386 examinations
AUC	0.96 (0.94–0.97)	0.95 (0.93–0.97)	0.97 (0.95–0.98)	0.97 (0.95–0.98)	0.98 (0.96–0.99)
AUC, no cast	0.94 (0.91–0.96)	0.93 (0.89–0.96)	0.94 (0.91–0.97)	0.95 (0.92–0.98)	0.96 (0.93–0.98)
Sensitivity	0.92 (0.90–0.94)	0.90 (0.86–0.93)	0.94 (0.91–0.97)	0.97 (0.94–0.99)	0.95 (0.92–0.97)
Sensitivity, no cast	0.86 (0.81–0.91)	0.83 (0.75–0.90)	0.90 (0.84–0.95)	0.94 (0.89–0.98)	0.90 (0.84–0.95)
Specificity	0.88 (0.84–0.92)	0.87 (0.81–0.93)	0.90 (0.84–0.95)	0.80 (0.73–0.87)	0.91 (0.86–0.96)
Specificity, no cast	0.89 (0.84–0.93)	0.87 (0.81–0.93)	0.90 (0.84–0.95)	0.81 (0.74–0.88)	0.92 (0.86–0.97)
Accuracy	0.91 (0.89–0.93)	0.89 (0.86–0.92)	0.93 (0.90–0.95)	0.92 (0.89–0.94)	0.94 (0.91–0.96)
Accuracy, no cast	0.88 (0.85–0.91)	0.85 (0.80–0.90)	0.90 (0.86–0.94)	0.87 (0.82–0.91)	0.91 (0.87–0.95)
PPV	0.95 (0.93–0.97)	0.94 (0.91–0.97)	0.96 (0.93–0.98)	0.92 (0.89–0.95)	0.96 (0.94–0.98)
PPV, no cast	0.87 (0.82–0.92)	0.85 (0.78–0.92)	0.89 (0.83–0.95)	0.82 (0.74–0.88)	0.91 (0.85–0.96)
NPV	0.83 (0.78–0.87)	0.79 (0.72–0.86)	0.87 (0.80–0.92)	0.91 (0.85–0.96)	0.88 (0.82–0.94)
NPV, no cast	0.88 (0.84–0.92)	0.85 (0.78–0.91)	0.91 (0.85–0.96)	0.94 (0.88–0.98)	0.91 (0.85–0.96)
LR+ , no cast	7.8 (5.35–11)	6.38 (3.91–10)	9.0 (5.12–16)	4.95 (3.35–7.3)	11 (5.95–21)
LR–, no cast	0.16 (0.11–0.22)	0.20 (0.13–0.30)	0.11 (0.06–0.20)	0.07 (0.03–0.16)	0.11 (0.06–0.20)

*p-a* postero-anterior, *AUC* area under the curve, *no cast* examinations in the test set without a cast (47% fracture), *PPV* positive predictive value, *NPV* negative predictive value, *LR*+ positive likelihood ratio, *LR− *negative likelihood ratio

For images with a cast but without a fracture, the model correctly assessed two out of the four examinations (eight images). For the remaining two examinations (four images), the DL model assessed one postero-anterior and one lateral radiograph as abnormal despite the absence of fracture (i.e., 6/8 images assessed correctly).

**Fig. 5:**
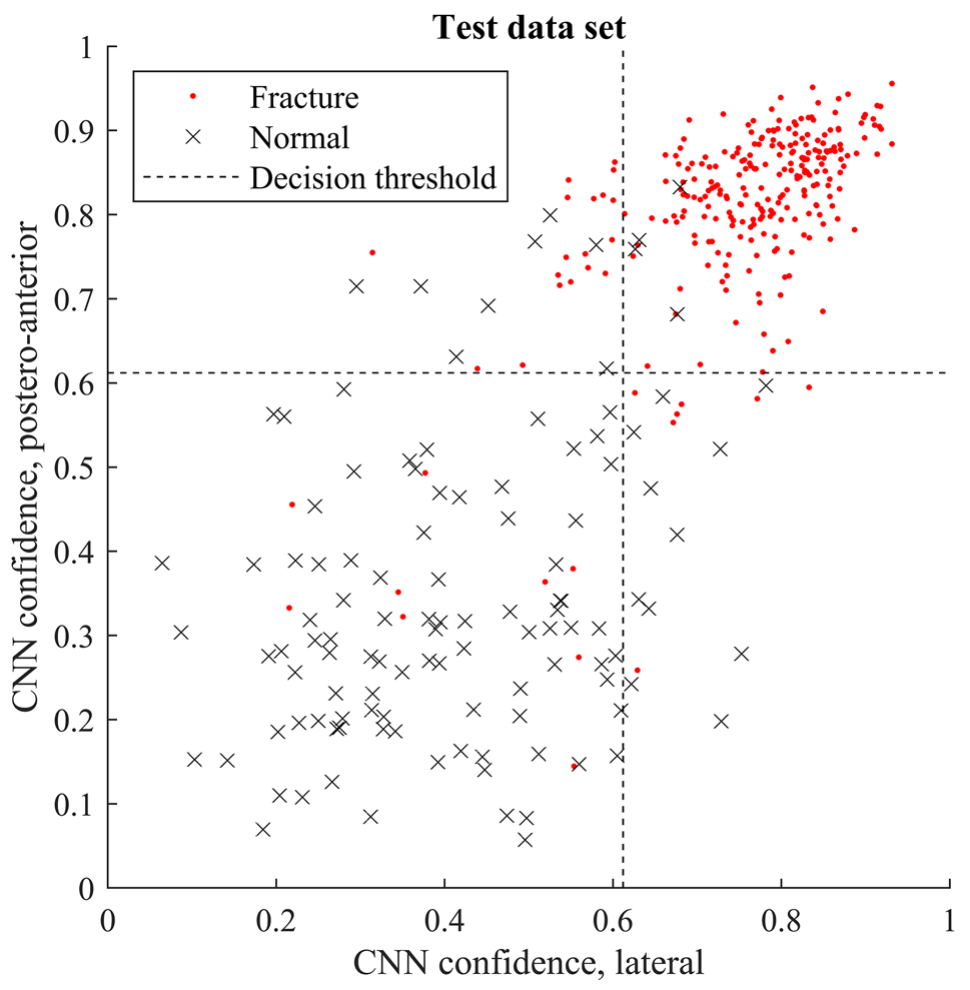
Deep learning model output values (confidences) for lateral and postero-anterior views for the test data set. The decision threshold (output confidence ≥ 0.61 operating point) based on validation data during training is indicated with the dotted lines

The model’s agreement on the lateral and postero-anterior views of the same wrist was 87%. The error rate for both lateral and postero-anterior predictions being incorrect was 3%. Figure 5 shows the output values for the lateral and posteroanterior views.

The intermediate CNN models’ performances were also tested to estimate how much the auxiliary network and the shift-and-average schemes affected the results. In the test set, the former improved the maximum test accuracy from 0.92 to 0.93. The latter improved the accuracy from 0.93 to 0.94.

Different radiograph system manufacturers performed similarly in our data as shown in Table 4, although a small number of radiographs taken with Carestream Health and GE Healthcare devices limit the generalizability of the results.

**Table 4:** Showing the results by radiograph system manufacturer

Manufacturer	True positive	False positive	True negative	False negative
Agfa	6	1	2	0
Carestream Health	1	0	1	0
Canon Inc	27	0	20	2
Fujifilm Corporation	47	0	20	5
GE Healthcare	4	0	0	0
Philips Medical Systems	35	1	11	1
Samsung Electronics	137	8	51	6
In total	257	10	105	14

## Discussion

Our results show that segmentation-based neural networks can be beneficial when assessing musculoskeletal trauma imaging. After training with 3399 images, the DL model could correctly identify 262 (96,7%) fractures and missed 9 (3,3%) fractures in a sample of 271 fractures.

Diagnostic errors in emergency rooms have been shown to cause patient harm and malpractice claims [4]. The overcrowding in emergency rooms may predispose patients to errors even more [19]. Thus, there is a demand for diagnostic aid and our model seems to perform well also with different scanner brands.

Previous studies have shown the feasibility of CNN in fracture detection. Olczak et al. reported results from hand, wrist, and ankle radiographs with the highest accuracy of 83% [20]. Gan et al. has also developed an AI model for fracture detection in wrist postero-anterior projections with a detection rate of AUC 0.96 [11]. Kim and MacKinnon reached a fracture detection of AUC 0.954 in lateral projection [21]. Thian et al. reported results for radius and ulna fractures reaching an AUC of 0.933 in lateral images and 0.918 in ap images with similar results also for fracture detection in radiographs with cast [12]. Lindsey et al. reported also on DRF detection and achieved AUC of 0.98 and showed increased clinician accuracy [22]. Our test set results are in line with the other publications.

A limitation of our study is the possibility of mislabeled ground truth and gold standard radiographs due to fractures not visible in wrist radiographs. These fractures are in an exact position and can only be reliably diagnosed with computed tomography (CT) or magnetic resonance imaging (MRI). While CT or MRI images were not available for this data set, the clinical importance of these fractures can be disputed and detecting them may not be useful at all.

Our study’s strengths were the representative sample of patients, expertise of test-set annotators, training data from six different hospitals, radiographs taken with systems from several different manufacturers, and the novel use of direct semantic segmentation as a basis for fracture detection.

In conclusion, our segmentation-based neural network performed well in our multi-hospital and multi-radiograph system manufacturer setting. Further research should include comparison of different algorithms and external validation. In the future, we think that meticulously developed and validated AI models will be able to assess the alignment and fragmentation of DRFs. Fracture detection is just the first, but necessary, step to aid clinicians improve the treatment of DRF patients.

## Supplement

In this supplement is described in more detail the CNN architecture and training.

### Model Training

**Fig. 6:**
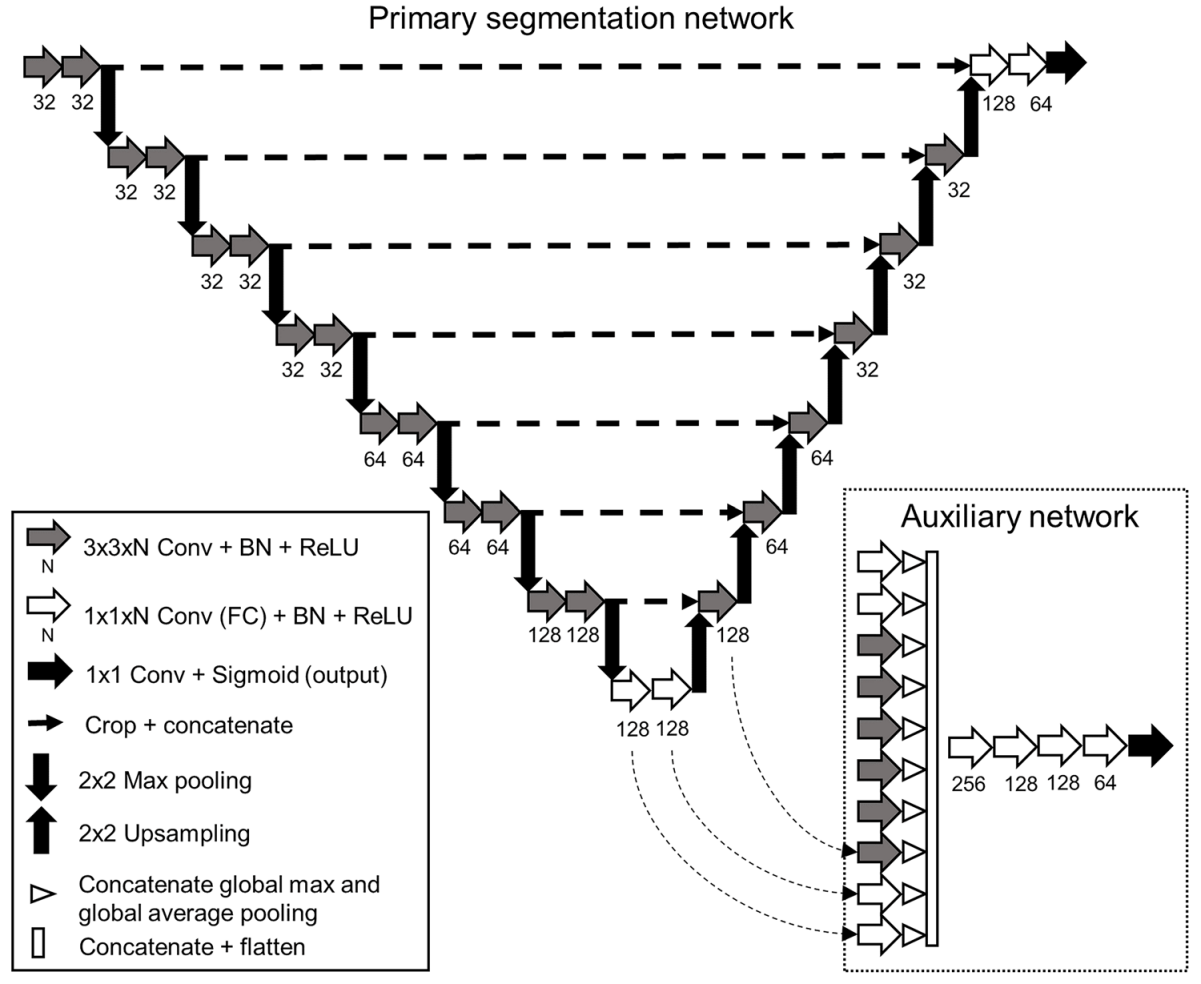
U-Net based convolutional neural network model consisted of 25 layers, seven max-pooling/up-sampling steps, and skip-connections between the matching resolution levels. Valid padding, 3 × 3 filters, batch normalization (BN), and rectified linear unit (ReLU) activation were used in the convolutional layers. Dropout layers were used prior to the three final convolutional layers. After five epochs, an auxiliary network was included in the training; global average and max pooling were applied to the outputs of the fully connected (FC) and the up-sampling pathway convolutional layers. Only the first three connections are shown in the figure (curved dashed arrow). The pooling layer outputs were concatenated and flattened and followed by five FC layers. The auxiliary network was used only during the second phase of the training. The output from the auxiliary network was not used in the inference (testing)

The segmentation model was based on U-Net and consisted of 25 layers with seven max-pooling/up-sampling layers with skip-connections between encoding and decoding pathways (Fig. 6) [13]. Training and inference were performed in a patch-based manner. An image was split into 130 × 130 pixel-sized sub-images and with appropriate overlapping extension at the edges (a larger input was required by the valid padding in the convolutional layers) and fed into the network. The outputs were stitched together at the end to produce the fracture confidence map for the whole image. We used Adam optimizer with a learning rate of 0.001 and binary cross entropy loss function in training. We performed validation on a randomly sampled 10% of the training data. Validation accuracy did not improve after five epochs (with or without lowering the learning rate).

At this point, we created an auxiliary network to encourage alternative decision pathways by applying global max and average pooling to the outputs of the bottom most and the succeeding convolutional layers. This was followed by concatenation and flattening and four fully connected layers, and a single output neuron with sigmoid activation. The combined model was further trained with a learning rate of 0.0025 and using the sum of the binary cross-entropy loss from the segmentation network output and the auxiliary network output. The training target for the auxiliary network was the binary choices if fracture drawing is present or is not present in the patch. After an additional three epochs, the validation (segmentation) accuracy did not further improve, and the resulting model was chosen for testing.

By utilizing the auxiliary network after initial training, we observed improved segmentation learning during the training phase. The second approach to improve accuracy was to repeat the inference with 26 pixel shifts in the horizontal and vertical directions and averaging the resulting 25 outputs. We chose this approach regardless of the increased processing time, as it resulted in improved validation accuracy.

## Data Availability

Under the terms of our Institutional Review Board approval, the retrospective data used in this study, from Helsinki University Hospital, cannot be released to protect patient confidentiality. Regarding the CNN training, the source code is available from the corresponding author upon reasonable request.
